# Excessive Weight Gain During Pregnancy Increased Ponoxarase 1 Level in Neonatal Cord Blood

**DOI:** 10.3390/antiox14010105

**Published:** 2025-01-17

**Authors:** Serhat Ege, Hasan Akduman, Ayşegül Aşır, Tuğcan Korak

**Affiliations:** 1 Department of Obstetrics and Gynecology, Medical Faculty, Dicle University, Diyarbakır 21280, Turkey; 2 Division of Neonatal Intensive Care Unit, Clinic of Pediatrics, Etlik City Hospital, Ankara 06170, Turkey; 3 Division of Pediatrics, Gazi Yaşargil Training and Research Hospital, Diyarbakır 21090, Turkey; 4 Department of Medical Biology, Faculty of Medicine, Kocaeli University, Kocaeli 41380, Turkey

**Keywords:** pregnancy, BMI, oxidative stress, umbilical cord, antioxidant

## Abstract

Maternal obesity is increasingly recognized as a risk factor for adverse fetal outcomes, primarily through its association with heightened oxidative stress. This study aimed to evaluate oxidative stress markers in umbilical cord blood of neonates born to obese mothers. Sixty-three pregnant women, who were of normal weight at the start of pregnancy but classified as obese at term, were included. Umbilical cord blood samples were collected immediately post-delivery and analyzed for serum oxidative stress markers (total oxidant status (TOS), total antioxidant status (TAS), paraoxanase (PON), aryl esterase, thiol, and catalase activities). Protein interaction networks were generated using Cytoscape (v3.10.3), and the overlapping proteins were further analyzed for functional annotations with ShinyGO (0.80). The top ten significantly enriched pathways were identified with a false discovery rate (FDR) threshold of <0.05. Significant associations were found between maternal BMI change and paraoxonase 1 (PON1) levels in umbilical cord blood, while no correlation was observed with other oxidative (total oxidant status) and antioxidant markers (total antioxidant status, aryl esterase, thiol, and catalase). Additionally, the correlation analysis showed a significant relationship between BMI change and fetal gestational age, but not with other demographic or clinical features. A total of 24 common protein interactors associated with PON1, obesity, and oxidative stress were identified. Functional annotation analysis revealed significant enrichment in antioxidant and oxidoreductase activities, along with pathways involved in insulin resistance, AGE-RAGE signaling, and atherosclerosis. Maternal obesity may specifically affect PON1 activity, potentially serving as a compensatory response to oxidative stress in neonates, suggesting PON1 as a possible biomarker for oxidative stress-related metabolic disturbances in neonates of obese mothers, with implications for monitoring and managing pregnancy outcomes in obese populations.

## 1. Introduction

Obesity is a nutritional disorder and is defined as abnormal or excessive fat accumulation that poses a risk to human health. Body mass index (BMI, kg/m^2^) is a measurement used to show nutritional data in adults. According to the World Health Organization (WHO), nutritional status is defined as BMI < 18.5 underweight, 18.5 ≤ BMI ≤ 24.9 normal weight, 25.0 ≤ BMI preobesity, and 30.0 ≤ obesity [1]. However, in the context of pregnancy, “maternal obesity” typically refers to women who are obese prior to conception or early in gestation, rather than those who only become obese at term [2,3]. According to the American College of Obstetricians and Gynecologists (ACOG), women with a normal pre-pregnancy BMI (18.5–24.9 kg/m^2^) are recommended to gain approximately 11.5–16 kg (25–35 lbs) over the course of pregnancy, and exceeding these guidelines is considered excessive gestational weight gain rather than pre-pregnancy maternal obesity [4,5,6]. Maternal weight gain during pregnancy is critical for the health of both the mother and the fetus. Inadequate prenatal weight gain can lead to fetal growth restriction (FGR), preterm birth, and miscarriage, while excessive weight gain (including becoming obese by term) has been associated with adverse maternal outcomes, prematurity, fetal death, and neonatal metabolic disturbances [7,8]. Studies have shown that children of overweight or obese mothers—whether the obesity is pre-existing or develops during gestation—have an increased risk of neural tube defects, limb anomalies, cleft palate and lip, and congenital heart defects compared to those of normal-weight mothers [9].

Oxidative stress is a molecular damage in which the imbalance between antioxidants and oxidants shifts towards the oxidant side and causes inflammation, endoplasmic reticulum stress, autophagy, and tissue damage. Fat accumulation is known to be an important factor of obesity, and obesity-induced oxidative stress has been shown to be associated with many diseases and to cause comorbidity [10,11]. Chardi et al. [12] recorded increased oxidative stress, increased malondialdehyde (MDA), and decreased superoxide dismutase (SOD) values in the adipose tissue of rats in an experimental high-fat-diet-induced obesity model. In another study, it was reported that consumption of high-fat diets increased the level of free fatty acids, caused b-oxidation in mitochondria, and caused an increase in reactive oxygen species (ROS) by providing intense electron flow [13]. In the blood of 724 patients with metabolic syndrome, oxidative stress parameters malondialdehyde (MDA) and total oxidant capacity (TAC) were found to be the highest, and superoxide dismutase (SOD) and thiol activities were found to be low. The authors stated that oxidative stress and obesity are closely related [14]. It was observed that high-fat and carbohydrate nutrition caused prolonged and greater oxidative stress and inflammation in obese individuals compared to normal-weight individuals [15].

Umbilical cord blood provides an important reflection of the intrauterine environment and biochemical status. Cord blood, which mediates a continuous exchange of nutrients and oxygen between the mother and the fetus, is an ideal biological material for assessing the metabolic and oxidative stress status of the fetus [16]. Cord blood can directly reflect the oxidative stress levels that the fetus is exposed to during the intrauterine period, especially in order to examine the effects of maternal obesity on the fetus. In addition, the fact that umbilical cord blood can be easily collected during birth and analyzed without affecting the health of the newborn makes cord blood important [17,18].

This study investigated the pregnant women who started with a normal BMI at the onset of pregnancy but exceeded the recommended weight gain during gestation, resulting in a BMI value that fell within the obese range by the end of pregnancy. The evaluation of oxidative stress markers in newborns may play an important role in understanding the effects of maternal excessive weight gain in the prenatal period on the fetus. The aim of this study was to reveal the effects of maternal excessive weight gain on serum oxidative stress markers in umbilical cord blood of newborns.

## 2. Materials and Methods

### 2.1. Study Design

This prospective study was conducted in Diyarbakır Gazi Yaşargil Training and Research Hospital between 1 January 2024 and 1 September 2024. Informed consent was obtained from all patients, and they participated in this study voluntarily. The ethics committee decision for this study was taken from the Gazi Yaşargil Clinical Research Ethical Board with the ethics committee number 2024/243. This study was conducted in accordance with the Helsinki Declaration. Sixty-three patients were included in this study. The number of patients in this study was determined according to Gpower (version 3.1) power analysis; effect size: 0.3, alpha error: 0.5, and power: 0.80.

### 2.2. Patient Collection

This study included 63 pregnant women who were of normal weight according to the BMI index at the beginning of pregnancy but gained excessive gestational weight according to the BMI index at the end of pregnancy. The pregnant women did not have any other systemic disease. The patients did not use any medication, cigarettes, alcohol, or drugs during pregnancy. All pregnancies were singletons, and no fetal anomalies or fetal deaths occurred. Women with fetal growth restriction, fetal anomalies, multiple pregnancies, preterm birth, and preterm premature rupture of the membranes were not included. The BMI index was determined according to WHO criteria [19]. Immediately following delivery, umbilical cord blood was collected into sterile, heparinized collection tubes. To minimize pre-analytical variability, all samples were processed within 30 min of collection. Samples were centrifuged at 3000× *g* for 10 min at 4 °C to separate plasma from cellular components. The plasma fractions were then carefully aliquoted into polypropylene tubes to avoid multiple freeze–thaw cycles and stored at −80 °C until analysis. Throughout sample handling, low-light conditions were maintained to prevent oxidation artifacts, and all laboratory equipment and reagents were maintained in accordance with the manufacturers’ guidelines. Prior to measurement, samples were thawed once on ice and mixed gently to ensure uniformity without introducing air bubbles. The use of standardized protocols for sample collection, processing, and storage was intended to preserve the integrity and stability of oxidative and antioxidant markers, thereby ensuring reliable and reproducible analytical results. Venous umbilical blood represents placental function since it flows from the placenta to the fetus. This blood can be used to evaluate markers associated with maternal–fetal transfer as well as placental function and nutrient delivery.

### 2.3. Laboratory Analysis

#### 2.3.1. Measurement of Total Oxidant Status (TOS)

Total oxidant status of serum was measured with a commercial kit (Rel Assay Diagnostics, Gaziantep, Turkey, catalog no: RL0024) via a colorimetric method, coefficient of variation%: 10; linearity: 0–33.5 mmol/L. Measuring was performed based on a novel automated measurement method by Erel et al. [20]. Briefly, oxidants in a sample oxidize a ferrous ion-o-dianisidine complex to a ferric ion, with glycerol enhancing the reaction. The ferric ion then reacts with xylenol orange in an acidic medium, forming a colored complex. The intensity of this color, measured spectrophotometrically, correlates with the sample’s total oxidant levels. Calibration is performed with hydrogen peroxide, and results are reported as micromolar hydrogen peroxide equivalents per liter (μmol H_2_O_2_ equiv./L). This method demonstrates high precision, with variability under 2%.

#### 2.3.2. Measurement of Total Antioxidant Status (TAS)

Total antioxidant status of serum was measured with a commercial kit (Rel Assay Diagnostics, Gaziantep, Turkey, catalog no: RL0017) via a colorimetric method, coefficient of variation%: 10; linearity: 0–2.75 mmol/L. Measuring was performed based on a novel automated measurement method by Erel et al. [21]. Briefly, free radical reactions were initiated via the Fenton reaction to produce hydroxyl radicals, with the rate monitored by the absorbance of colored dianisidyl radicals. The sample’s antioxidant effect against these radicals was measured, and the method was run on an Aeroset^®^ automated analyzer (Abbott, Abbott Park, IL, USA). The assay showed high precision, with intra- and inter-assay variation coefficients below 3%, and results were reported as TAS in mmol Trolox equivalents per liter (mmol Trolox equiv./L).

#### 2.3.3. Measurement of Paraoxanase 1 and Aryl Esterase Activities

Paraoxonase 1 and aryl esterase levels were measured by a commercial kit (Rel Assay Diagnostics, Gaziantep, Turkey). Paraoxonase 1 (PON1) activity was assessed by measuring the hydrolysis rate of paraoxon, a toxic organophosphate, at 412 nm and 37 °C. The quantity of p-nitrophenol produced was calculated using a molar absorptivity coefficient of 17,013 M^−1^ cm^−1^ at pH 8, and results were expressed in units per liter (U/L) of serum. For aryl esterase activity, phenylacetate served as the substrate, and the reaction’s absorbance increase at 270 nm at 37 °C was recorded. Enzymatic activity was determined with a molar absorptivity of 1310 M^−1^ cm^−1^ for phenol, defining one unit as the amount of enzyme that generates 1 μmol phenol per minute, with results expressed as kU/L serum [22,23].

#### 2.3.4. Measurement of Thiol Level

Thiol levels were spectrophotometrically measured using the automatic method described by Erel et al. [24]. Thiol/disulfide homeostasis tests involved reducing disulfide bonds to generate free thiol groups. Unused sodium borohydride reductant was neutralized with formaldehyde, and total thiol levels were measured after reaction with DTNB. The difference between total and native thiol levels, divided by two, provided the dynamic disulfide amount. Ratios of disulfide/total thiol, native thiol/total thiol, and disulfide/native thiol were then calculated to assess thiol/disulfide balance.

#### 2.3.5. Measurement of Catalase Activity

Catalase activity was measured spectrophotometrically following the Koroliuk et al. [25] method. A 10 μL sample was incubated with hydrogen peroxide in a Tris-HCl buffer (pH 7) for 10 min, and the reaction was stopped with ammonium molybdate. The resulting yellow complex was measured at 410 nm. One unit of catalase activity equaled the enzyme amount needed to decompose 1 μmol of H_2_O_2_ per minute.

### 2.4. Network and Pathway Enrichment Analysis of PON1 in Obesity and Oxidative Stress

To explore the molecular mechanisms potentially affected by the significant association of paraoxonase 1 (PON1) levels (both total and free) with oxidative stress in maternal obesity, network and functional annotation analyses were conducted. Protein–protein interaction (PPI) networks were constructed in Cytoscape (v3.10.3) using the STRING database for obesity, oxidative stress, and PON1, with settings of a maximum interaction count of 200 proteins and a medium confidence threshold (0.4) [26,27]. The intersecting proteins from these three networks were subsequently analyzed. The resulting common proteins underwent gene ontology (GO), molecular function (MF), and Kyoto Encyclopedia of Genes and Genomes (KEGG) pathway annotations using the ShinyGO 0.80 platform (http://bioinformatics.sdstate.edu/go80/ accessed on 11 October 2024). Pathways were ranked based on fold enrichment values with a false discovery rate (FDR) threshold of <0.05, and the top ten pathways were represented [28,29].

### 2.5. Statistical Analysis

All statistical analyses were performed using IBM SPSS for Windows version 29.0 (IBM Corp., Armonk, NY, USA). Kolmogorov–Smirnov’s and Shapiro–Wilk’s tests were used to assess the normality assumption. Continuous variables were presented with mean ± standard deviation or median and interquartile range (IQR). Categorical variables were summarized as counts and percentages. Comparisons between groups were carried out using the Mann–Whitney U test to observe statistical significance with two parameters with respect to BMI (male vs. female, AGA vs. LGA, etc.). Associations between continuous variables were examined by Spearman’s correlation analyses. A *p*-value < 0.05 was considered statistically significant.

## 3. Results

### 3.1. Demographic and Clinical Properties of Patients

Demographic properties of pregnant women and their babies were presented in Table 1. While women were normal weight at the onset of pregnancy, they were overweight at the end of the pregnancy. The numerical change and percentage change in BMI showed that women were overweight at the end of pregnancy. Fetal birth weight and APGAR scores indicated healthy babies.

Table 2 shows clinical features of pregnant women and their supplementation during pregnancy. Most babies were born with average weight.

### 3.2. Levels of Antioxidant and Oxidant Markers in Patients

Oxidant and antioxidant markers measured from umbilical cord blood of neonates were shown in Table 3.

### 3.3. Increased BMI Is Associated with Fetal Gestational Age

The relationship of demographic and clinical parameters with BMI was shown in Table 4. There was no statistical significance between gender, abortus, labor delivery, and folic acid supplementation and BMI change, while fetal gestational age was significantly associated with BMI change. The lack of statistical significance between BMI change and variables such as gender, abortus, labor delivery, and folic acid supplementation suggests that these factors may not substantially influence BMI fluctuations during pregnancy. However, the significant association between fetal gestational age and BMI change highlights that as pregnancy progresses, weight gain patterns in the mother may shift, potentially due to increased physiological demands and metabolic changes needed to support fetal development.

### 3.4. Increased BMI Is Correlated with PON1

The correlation analysis of demographic and clinical parameters with BMI was shown in Table 5. Birth week, maternal age, gravida, parity, APGAR scores, and fetal birth weight were not statistically correlated with change in BMI. The increase in weight was not associated with BMI change. Among antioxidants, only PON1 and soluble PON1 were significantly correlated with BMI change; however, TAS, aryl esterase, thiol, and catalase did not show statistical significance with BMI change. Oxidative stress marker TOS was also not significantly associated with BMI change. Similar to other demographic and clinical parameters in Table 4, the absence of a statistically significant correlation between BMI change and factors such as birth week, maternal age, gravida, parity, APGAR scores, and fetal birth weight implies that BMI change in pregnancy can be driven more by metabolic or physiological adjustments rather than by birth timing, maternal age, or neonatal outcomes. The lack of significant correlation between most antioxidants (TAS, aryl esterase, thiol, and catalase) and BMI change, as well as the oxidative stress marker TOS, indicates that BMI increase may not uniformly alter the oxidative or antioxidant profiles in maternal or fetal systems. This highlights that oxidative stress and antioxidant capacity do not necessarily escalate with higher BMI, which might suggest a complex or nonlinear relationship between BMI and oxidative status during pregnancy. The significant correlation found only with PON1 and soluble PON1 may suggest that these enzymes play a more specific role in metabolic responses to BMI change.

### 3.5. Relationship of Birth Weight, Gender, and Oxidative Stress Markers

The median birth weight for females was 3115 g (2863–3620 g), and 3350 g (2990–3560 g) for males. No significant difference in birth weight was found between the sexes (*p* = 0.368). In addition, no significant difference in oxidative stress was found between the sexes (*p* > 0.05 for all) (Table 6).

Although there was no difference in birth weight between the sexes, the association between birth weight and oxidative stress varied by sex. No significant correlation was found between birth weight and oxidative stress in males, while a moderate positive correlation was significantly found between birth weight and PON1 (r = 0.412, *p* = 0.029) and soluble PON1 (r = 0.406, *p* = 0.032) in females (Table 7).

### 3.6. Functional Annotation Analysis of PON1-Associated Interactors in Oxidative Stress and Obesity

**Figure 1:**
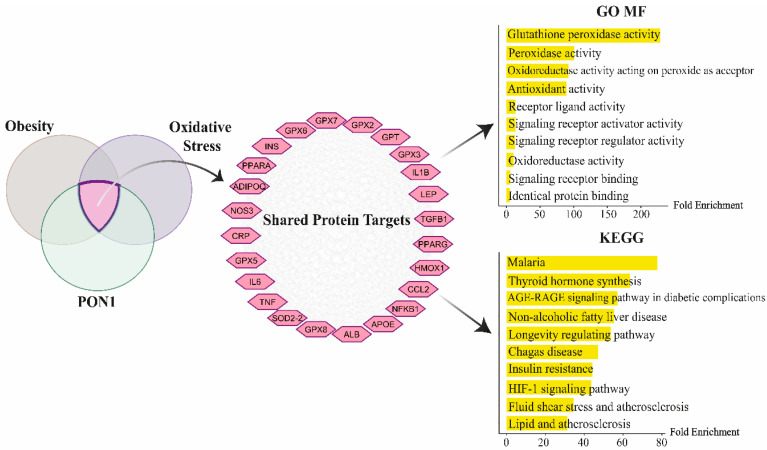
Protein–protein interaction network and functional enrichment analysis of PON1 interactors associated with obesity and oxidative stress. Functional annotations using gene ontology (GO), molecular function (MF), and Kyoto Encyclopedia of Genes and Genomes (KEGG) reveal the top ten significant enrichments based on 24 shared protein targets.

The PPI network analysis revealed 24 PON1 protein interactors associated with obesity and oxidative stress. These interactors included interleukin 6 (IL6), C-reactive protein (CRP), insulin (INS), interleukin 1 beta (IL1B), transforming growth factor beta 1 (TGFB1), heme oxygenase 1 (HMOX1), peroxisome proliferator-activated receptor alpha (PPARA), C-C motif ligand 2 (CCL2), glutathione peroxidase 2 (GPX2), GPX3, GPX5, GPX6, GPX7, GPX8, glutamic–pyruvic transaminase (GPT), albumin (ALB), peroxisome proliferator-activated receptor gamma (PPARG), nuclear factor kappa B subunit 1 (NFKB1), nitric oxide synthase 3 (NOS3), tumor necrosis factor (TNF), apolipoprotein E (APOE), superoxide dismutase 2 (SOD2), adiponectin (ADIPOQ), and leptin (LEP). GO MF analysis of these intersecting proteins revealed significant enrichment in glutathione peroxidase activity, peroxidase activity, oxidoreductase activity acting on peroxide as an acceptor, and antioxidant activity. KEGG pathway enrichment analysis identified significant pathways, including the advanced glycation end-products (AGEs) and their receptors (RAGE) (AGE-RAGE) signaling pathway in diabetic complications, insulin resistance, hypoxia-inducible factor 1 (HIF-1) signaling pathway, non-alcoholic fatty liver disease, lipid and atherosclerosis, and fluid shear stress and atherosclerosis (Figure 1).

## 4. Discussion

In this study, our population represents cases of excessive gestational weight gain rather than traditional maternal obesity established before pregnancy. This distinction is important because excessive weight gain during pregnancy can also lead to adverse maternal and fetal outcomes. TOS and TAS and enzyme activities such as paraoxonase 1, aryl esterase, thiol, and catalase were examined to evaluate the oxidative stress status in babies born to pregnant women with high BMI. Statistical analysis results revealed that especially paraoxonase 1 and sPON1 enzyme activities showed a significant correlation with BMI change, but no significant difference was found in other antioxidant enzyme activities.

Reactive oxygen species (ROS) are unstable molecules that cause cellular damage and deteriorate cell functions. Excessive ROS production causes oxidative stress in the cell, while the cell increases the production of antioxidant enzymes (catalase, SOD, GSH, etc.) as a defense [30]. There are studies showing a positive relationship between obesity (fat accumulation) and oxidative stress in humans [31].

The relationship between oxidative stress in the perinatal period and pregnancy and neonatal diseases has been shown [32]. In 20 umbilical cord blood samples of newborns of preeclamptic pregnant women, the level of oxidative stress markers was found to be higher than in normotensive patients [33]. In another study, it was observed that high MDA and low TAS values in newborn blood samples of preeclamptic pregnant women were correlated with adverse neonatal outcomes [34]. It was observed that oxidative stress indices were high in the blood serum of women who gave birth with intrauterine growth restriction (IGUR) [35]. In a proteomic study examining the differential expression of different proteins in obese placenta, it was found that ferritin and mitochondrial proteins were under-expressed, which may induce the oxidative stress mechanism [36]. It has been claimed that oxidative stress levels are high in placentas and neonates of obese people, and that this is related to metabolic alterations and dysfunction [37]. Jantape et al. reported that maternal obesity is associated with placental and fetal oxidative stress [38]. In our study, demographic and clinical properties were not significantly correlated with BMI change; this could be because these variables either have limited or indirect effects on maternal weight gain or fat accumulation during gestation. Fetal gestational age might influence BMI change more directly, as it reflects the duration and intensity of these physiological adjustments, which are critical to sustaining pregnancy and fetal growth.

For serum markers, TAS, TOS, aryl esterase, free thiol, and catalase enzymes were correlated with increasing BMI difference and percentage change, but this correlation was not statistically significant. Paraoxonase 1 and soluble paraoxonase 1 enzyme levels were significantly correlated with increasing BMI and percentage BMI change.

This highlights that oxidative stress and antioxidant capacity do not necessarily escalate with higher BMI, which might suggest a complex or nonlinear relationship between BMI and oxidative status during pregnancy. The significant correlation found only with paraoxonase 1 and soluble paraoxonase 1 may suggest that these enzymes play a more specific role in metabolic responses to BMI change. Since paraoxonase enzymes are known to protect against lipid peroxidation, their activity may increase as a compensatory response to elevated oxidative stress that could accompany higher BMI. This unique association underscores paraoxonase’s potential role as a marker of metabolic adaptation in pregnant women with increased BMI, differentiating it from other antioxidant markers that appear less sensitive to BMI-related metabolic changes.

An intriguing aspect of our findings is the observed sex-specific differences in the relationship between fetal birth weight and oxidative stress markers, particularly the significant positive correlation between birth weight and paraoxonase 1 activity in female neonates, which was not evident in their male counterparts [39,40]. Previous studies suggest that female fetuses have a higher baseline antioxidant capacity compared to males, which might explain the positive correlation between birth weight and PON1 levels in females. PON1 is a known protective enzyme against lipid peroxidation, and its increased activity in females could be a compensatory mechanism to mitigate oxidative stress during development. Studies have documented that male fetuses are more vulnerable to adverse outcomes under suboptimal intrauterine conditions, including oxidative stress, due to a less adaptive response system. This aligns with the lack of significant correlations in males observed in this study, underscoring the need for further research into sex-specific fetal programming [41,42,43]. These findings highlight the potential influence of fetal sex on the relationship between birth weight and oxidative stress, particularly emphasizing a sex-specific association with paraoxonase 1 and soluble paraoxonase 1 in female neonates. This contrasts with the absence of such correlations in male neonates, suggesting sex-specific differences in the neonatal oxidative stress response to maternal BMI changes. Identifying sex-specific variations in oxidative stress markers such as PON1 can help tailor antenatal interventions. For instance, promoting maternal antioxidant intake might have differential benefits based on fetal sex, enhancing protective mechanisms where needed.

The pathway and functional annotation analyses revealed significant enrichment in processes related to antioxidant defense, such as glutathione peroxidase activity, peroxidase activity, and oxidoreductase activity acting on peroxide. These findings underscore the potential role of PON1 in modulating oxidative stress, suggesting that it may be a key enzyme involved in the oxidative imbalance observed in the fetus of obese mothers, highlighting its potential as a marker for metabolic disturbances induced by maternal obesity. In line with these findings, one study demonstrated that PON1 activity influences infant development and birth weight, suggesting that PON1 enzyme levels in mothers could serve as a marker to predict the risk of low birth weight in offspring [30]. Further supporting the importance of PON1, it was supported that impaired HDL cholesterol efflux and PON1 activity in cord blood, along with increased maternal PON1 and apoM levels early in pregnancy, are associated with the risk of gestational hypertension. This further reinforces PON’s relevance in both metabolic and vascular health in mothers and their offspring [44]. Our study’s KEGG results align with these findings, identifying key pathways linked to metabolic and cardiovascular health, including the AGE-RAGE signaling pathway, insulin resistance, and non-alcoholic fatty liver disease. The AGE-RAGE axis, known for inducing oxidative stress and vascular damage, plays a crucial role in compromising both maternal and fetal health [45], while pathways related to lipid metabolism and atherosclerosis are key contributors to maternal obesity’s pathophysiology, linking lipid disturbances to adverse pregnancy outcomes and long-term cardiovascular risks for both mothers and offspring [46,47]; additionally, maternal obesity is a significant driver of insulin resistance, highlighting the relevance of the metabolic pathways identified in our analysis [48]. Moreover, the HIF-1 signaling pathway, which plays a critical role in cellular responses to hypoxia, was revealed to be suppressed in maternal obesity [49]. This suppression exacerbates oxidative stress and inflammation, thereby further compounding the metabolic disturbances associated with maternal obesity [50,51]. Together, these findings suggest that PON1 may be a key factor in understanding the metabolic disruptions associated with maternal obesity, offering potential insights into both maternal and fetal health.

This study has some limitations. Our data were collected at a single time point (at delivery). This design precludes drawing causal inferences about the relationships among maternal BMI, oxidative stress markers, and neonatal outcomes. Longitudinal follow-up or prospective studies would be necessary to establish directionality and causality. Although we excluded patients with major comorbidities and fetal anomalies, other potential confounders—such as maternal nutrition, physical activity, genetic predispositions, environmental exposures, and subtle metabolic variations—were not comprehensively assessed. Such factors could influence both maternal BMI and neonatal oxidative stress profiles. The relatively modest sample size may limit the statistical power to detect subtle differences and the generalizability of our findings. Minor variations in sample collection, handling, and storage, the stability and sensitivity of certain oxidative stress and antioxidant enzymes could potentially affect the accuracy and reproducibility of our measurements.

## 5. Conclusions

Our findings highlight the significant role of PON1 in modulating oxidative stress, emphasizing its potential as a biomarker for metabolic disturbances associated with maternal obesity. In addition, our in silico analysis identified several key metabolic and cardiovascular pathways related to PON1, including AGE-RAGE signaling, lipid metabolism, and insulin resistance. These insights contribute to a deeper understanding of the mechanisms underlying adverse pregnancy outcomes and long-term health risks for both mothers and offspring.

## Figures and Tables

**Table 1:** Maternal and fetal demographic properties.

Parameters	Value (n = 63)
Maternal age, mean ± SD, years	28.42 ± 5.90
Gravida, median (IQR) no unit	2 (1–2)
Parity, median (IQR) no unit	2 (1–2)
Birth week, mean ± SD	39.02 ± 1.13
Maternal height, median (IQR), cm	165 (160–167)
Weight at the onset of pregnancy, median (IQR), kg	60 (56–68)
Weight at the end of pregnancy, median (IQR), kg	74 (67–81)
BMI at the onset of pregnancy, median (IQR), kg/m^2^	22.3 (21–24.4)
BMI at the end of pregnancy, median (IQR), kg/m^2^	27.2 (25.3–28.6)
Change in BMI, median (IQR) kg/m^2^	4.6 (3.7–5.2)
Percentage Change in BMI, median (IQR)	19.23 (16.27–23.39)
Fetal birth weight, median (IQR) gr	3240 (2950–3590)
APGAR score at first minute, median (IQR)	9 (8–9)
APGAR score at fifth minute, median (IQR)	10 (9–10)

**Table 2:** Pregnancy-related maternal clinical features.

Clinical Features (n = 63)	Subtype	n (%)
Fetal gestational age	AGA	47 (85.5)
	LGA	7 (12.7)
	SGA	1 (1.8)
Fetal gender	Female	28 (50.9)
	Male	27 (49.1)
Maternal blood type	A (+)	14 (38.9)
	0 (+)	11 (30.6)
	B (+)	5 (13.9)
	Ab (+)	3 (8.3)
	A (−)	1 (2.8)
	B (−)	1 (2.8)
	0 (−)	1 (2.8)
Smoking	No	50 (92.6)
	Yes	4 (7.4)
Consanguineous marriage	No	51 (94.4)
	Yes	3 (5.6)
Pregnancy complication	None	51 (94.4)
	Yes	3 (5.6)
Delivery method	Spontaneous vaginal delivery	19 (33.9)
	C/S	37 (66.1)
Abortus	No	46 (83.6)
	Yes	9 (16.4)
Curettage	No	51 (92.7)
	Yes	4 (7.3)
Premature rupture of membrane	None	51 (92.7)
Chorioamnionitis	None	56 (100)
Folic acid supplementation	Yes	19 (35.8)
	No	34 (64.2)
Labor indication	Recurrent	18 (50)
	Prolonged	6 (16.7)
	Others	5 (13.9)
	Social	4 (11.1)
	Breech	3 (8.3)
Neonatal Resuscitation	Yok	52 (96.3)
	Var	2 (3.7)

AGA: average for gestational age; LGA: large for gestational age; SGA: small for gestational age; C/S: C section.

**Table 3:** The serum levels of oxidant and antioxidant makers in umbilical cord blood of neonates.

Markers	Level
TAS, median (IQR)	2.11 (1.98–2.27)
TOS, median (IQR)	1.27 (0.47–2.63)
PON1, median (IQR)	55.1 (31.2–100.4)
Soluble sPON1, median (IQR)	106.1 (48.6–275.9)
Aryl esterase, median (IQR)	45.7 (36.9–70.7)
Thiol, mean ± SD	203.98 ± 28.26
Catalase, median (IQR), catalase	69.82 (48.77–107.02)

TOS: total oxidant status; TAS: total antioxidant status; PON: paraoxanase; SD: standard deviation; IQR: interquartile range.

**Table 4:** Statistical significance between demographic parameters and BMI.

		Change in BMI Median (IQR)	% Change in BMI Median (IQR)
Gender	Female	4.65 (4.03–5.48)	19.99 (16.69–24.97)
	Male	4.7 (3.7–5.2)	19.47 (16.23–23.98)
	*p* *	0.717	0.946
Fetal gestational age	AGA	4.5 (3.7–5.1)	19.05 (16.23–22.95)
	LGA	6.1 (4.7–7)	28.77 (22.82–34.48)
	*p* *	0.035	0.002
Abortus	No	4.65 (3.85–5.18)	19.64 (16.46–23.1)
	Yes	5.1 (3–6.25)	22.41 (13.79–30.86)
	*p* *	0.657	0.539
Delivery	Vaginal	4.6 (3.7–5.1)	19.23 (16.23–22.82)
	C/S	4.7 (3.95–5.75)	19.82 (17.3–24.74)
	*p* *	0.291	0.528
Folic acid supplementation	No	4.55 (3.7–5.03)	18.9 (16.23–23.1)
	Yes	5.1 (4–5.8)	22.18 (18.96–26.01)
	*p* *	0.145	0.201

AGA: average for gestational age; LGA: large for gestational age; C/S: C section; *: Mann–Whitney U test; IQR: interquartile range.

**Table 5:** Correlation analysis between clinical features, oxidative stress markers, and BMI.

Features		Change in BMI	% Change in BMI
Birth week	r	0.055	0.036
	*p*	0.689	0.796
Maternal age	r	0.087	0.154
	*p*	0.529	0.263
Gravida	r	−0.072	−0.027
	*p*	0.600	0.846
Parity	r	−0.170	−0.133
	*p*	0.214	0.332
Apgar score first minute	r	−0.038	−0.015
	*p*	0.784	0.911
Apgar score fifth minute	r	0.023	0.045
	*p*	0.869	0.746
Fetal birth weight for male offspring	r	0.396	0.428
	*p*	0.041	0.026
Fetal birth weight for female offspring	r	0.094	0.100
	*p*	0.634	0.614
TAS	r	0.002	−0.047
	*p*	0.990	0.713
TOS	r	0.033	0.012
	*p*	0.797	0.929
PON1	r	−0.273	−0.306
	*p*	0.030	0.015
Soluble PON1	r	−0.256	−0.286
	*p*	0.043	0.023
Aryl esterase	r	−0.168	−0.175
	*p*	0.187	0.171
Thiol	r	−0.105	−0.175
	*p*	0.414	0.169
Catalase	r	0.163	0.109
	*p*	0.201	0.396

TAS: total antioxidant status; TOS: total oxidant status; PON: paraoxanase; r: Spearman’s correlation coefficient.

**Table 6:** Comparison of fetal birth weight and oxidative stress between sexes.

	Male (n = 27) Median (IQR)	Female (n = 28) Median (IQR)	*p* *
Birth weight	3350 (2990–3560)	3115 (2862–3620)	0.368
TAS	2.08 (1.97–2.2)	2.07 (1.95–2.25)	0.906
TOS	1.27 (0.38–1.92)	1.26 (0.52–2.61)	0.533
PON1	34.2 (30.8–92.1)	50.45 (29.93–99.93)	0.511
soluble PON1	64.3 (47.6–239.4)	112 (45.9–289)	0.533
Aryl esterase	42.1 (33.4–70.7)	45.5 (35.93–60.43)	0.866
Thiol	209 (181.6–222.9)	201 (175.08–230.25)	0.801
Catalase	59.65 (44.56–110.53)	81.58 (49.12–126.5)	0.359

TAS: total antioxidant status; TOS: total oxidant status; PON: paraoxanase; *: Mann–Whitney U test; IQR: interquartile range.

**Table 7:** Association between fetal birth weight and oxidative stress for each gender.

		Male	Female
TAS	0.214	r	0.052
	0.283	*p*	0.794
TOS	0.186	r	0.005
	0.353	*p*	0.979
PON1	−0.238	r	0.412
	0.233	*p*	0.029
Soluble PON1	−0.220	r	0.406
	0.269	*p*	0.032
Aryl esterase	−0.267	r	0.321
	0.178	*p*	0.096
Thiol	0.054	r	−0.022
	0.789	*p*	0.912
Catalase	0.240	r	−0.192
	0.229	*p*	0.328

TAS: total antioxidant status; TOS: total oxidant status; PON: paraoxanase; r: Spearman’s correlation coefficient.

## Data Availability

Data are available upon request. The commercial kits were purchases from Rel Assay Diagnostics (https://www.relassay.com/products.html accessed on 15 February 2024).

## References

[1] Nuttall F.Q. (2015). Body Mass Index: Obesity, BMI, and Health: A Critical Review. Nutr. Today.

[2] Institute of Medicine (IOM), National Research Council (2009). Weight Gain During Pregnancy: Reexamining the Guidelines.

[3] American College of Obstetricians and Gynecologists (ACOG) Committee Opinion No (2013). 548. Weight Gain During Pregnancy. Obstet. Gynecol..

[4] World Health Organization (WHO) (2000). Obesity: Preventing and Managing the Global Epidemic.

[5] Cnattingius S., Villamor E., Johansson S., Bonamy A.-K.E., Persson M., Wikström A.-K., Granath F. (2013). Maternal obesity and risk of preterm delivery. JAMA J. Am. Med. Assoc..

[6] Stotland N.E., Cheng Y.W., Hopkins L.M., Caughey A.B. (2006). Gestational weight gain and adverse neonatal outcome among term infants. Obstet. Gynecol..

[7] Addo V.N. (2010). Body Mass Index, Weight Gain during Pregnancy and Obstetric Outcomes. Ghana Med. J..

[8] Paredes C., Hsu R.C., Tong A., Johnson J.R. (2021). Obesity and Pregnancy. NeoReviews.

[9] Catalano P.M., Shankar K. (2017). Obesity and pregnancy: Mechanisms of short term and long term adverse consequences for mother and child. BMJ.

[10] Li H., Ren J., Li Y., Wu Q., Wei J. (2023). Oxidative stress: The nexus of obesity and cognitive dysfunction in diabetes. Front. Endocrinol..

[11] Furukawa S., Fujita T., Shimabukuro M., Iwaki M., Yamada Y., Nakajima Y., Nakayama O., Makishima M., Matsuda M., Shimomura I. (2004). Increased oxidative stress in obesity and its impact on metabolic syndrome. J. Clin. Investig..

[12] Charradi K., Elkahoui S., Limam F., Aouani E. (2013). High-fat diet induced an oxidative stress in white adipose tissue and disturbed plasma transition metals in rat: Prevention by grape seed and skin extract. J. Physiol. Sci..

[13] Tan B.L., Norhaizan M.E., Liew W.P. (2018). Nutrients and Oxidative Stress: Friend or Foe?. Oxid. Med. Cell Longev..

[14] Jakubiak G.K., Osadnik K., Lejawa M., Osadnik T., Goławski M., Lewandowski P., Pawlas N. (2021). “Obesity and Insulin Resistance” Is the Component of the Metabolic Syndrome Most Strongly Associated with Oxidative Stress. Antioxidants.

[15] Patel C., Ghanim H., Ravishankar S., Sia C.L., Viswanathan P., Mohanty P., Dandona P. (2007). Prolonged reactive oxygen species generation and nuclear factor-kappaB activation after a high-fat, high-carbohydrate meal in the obese. J. Clin. Endocrinol. Metab..

[16] Dutta S., Ruden D.M. (2024). Heavy Metals in Umbilical Cord Blood: Effects on Epigenetics and Child Development. Cells.

[17] Kindwall-Keller T.L., Ballen K.K. (2020). Umbilical cord blood: The promise and the uncertainty. Stem Cells Transl. Med..

[18] Zhou L., McDonald C., Yawno T., Jenkin G., Miller S., Malhotra A. (2022). Umbilical Cord Blood and Cord Tissue-Derived Cell Therapies for Neonatal Morbidities: Current Status and Future Challenges. Stem Cells Transl. Med..

[19] Khanna D., Peltzer C., Kahar P., Parmar M.S. (2022). Body Mass Index (BMI): A Screening Tool Analysis. Cureus.

[20] Erel O. (2005). A new automated colorimetric method for measuring total oxidant status. Clin. Biochem..

[21] Erel O. (2004). A novel automated direct measurement method for total antioxidant capacity using a new generation, more stable ABTS radical cation. Clin. Biochem..

[22] Seres I., Paragh G., Deschene E., Fulop T., Khalil A. (2004). Study of factors influencing the decreased HDL associated PON1 activity with aging. Exp. Gerontol..

[23] Haagen L., Brock A. (1992). A new automated method for phenotyping arylesterase (EC 3.1.1.2) based upon inhibition of enzymatic hydrolysis of 4-nitrophenyl acetate by phenyl acetate. Eur. J. Clin. Chem. Clin. Biochem..

[24] Erel O., Neselioglu S. (2014). A novel and automated assay for thiol/disulphide homeostasis. Clin. Biochem..

[25] Koroliuk M.A., Ivanova L.I., Maĭorova I.G., Tokarev V.E. (1988). A method of determining catalase activity. Lab. Delo.

[26] Ayaz H., Aşır F., Korak T. (2024). Skimmianine Showed Neuroprotection against Cerebral Ischemia/Reperfusion Injury. Curr. Issues Mol. Biol..

[27] Aşır F., Duran S.Ç., Afşin M., Duran E., Korak T., Şahin F. (2024). Investigation of Vitamin D Levels in Men with Suspected Infertility. Life.

[28] Keşim D.A., Aşır F., Ayaz H., Korak T. (2024). The Effects of Ellagic Acid on Experimental Corrosive Esophageal Burn Injury. Curr. Issues Mol. Biol..

[29] Aşır F., Özalp Z., Yülek Ö.U., Erdemci F., Korak T., Taş F. (2024). CITED1 expression in odontogenic cysts. BMC Oral Health.

[30] Jomova K., Raptova R., Alomar S.Y., Alwasel S.H., Nepovimova E., Kuca K., Valko M. (2023). Reactive oxygen species, toxicity, oxidative stress, and antioxidants: Chronic diseases and aging. Arch. Toxicol..

[31] Keaney Jr J.F., Larson M.G., Vasan R.S., Wilson P.W.F., Lipinska I., Corey D., Massaro J.M., Sutherland P., Vita J.A., Benjamin E.J. (2003). Obesity and systemic oxidative stress: Clinical correlates of oxidative stress in the Framingham study. Arterioscler. Thromb. Vasc. Biol..

[32] Longini M., Belvisi E., Proietti F., Bazzini F., Buonocore G., Perrone S. (2017). Oxidative Stress Biomarkers: Establishment of Reference Values for Isoprostanes, AOPP, and NPBI in Cord Blood. Mediat. Inflamm..

[33] Howlader M.Z., Parveen S., Tamanna S., Khan T.A., Begum F. (2009). Oxidative stress and antioxidant status in neonates born to pre-eclamptic mother. J. Trop. Pediatr..

[34] Namdev S., Bhat V., Adhisivam B., Zachariah B. (2014). Oxidative stress and antioxidant status among neonates born to mothers with pre-eclampsia and their early outcome. J. Matern. Neonatal Med..

[35] Karowicz-Bilinska A., Kędziora-Kornatowska K., Bartosz G. (2007). Indices of oxidative stress in pregnancy with fetal growth restriction. Free. Radic. Res..

[36] Oliva K., Barker G., Riley C., Bailey M.J., Permezel M., Rice G.E., Lappas M. (2012). The effect of pre-existing maternal obesity on the placental proteome: Two-dimensional difference gel electrophoresis coupled with mass spectrometry. J. Mol. Endocrinol..

[37] Malti N., Merzouk H., Merzouk S.A., Loukidi B., Karaouzene N., Malti A., Narce M. (2014). Oxidative stress and maternal obesity: Feto-placental unit interaction. Placenta.

[38] Jantape T., Kongwattanakul K., Arribas S.M., Rodríguez-Rodríguez P., Iampanichakul M., Settheetham-Ishida W., Phuthong S. (2024). Maternal Obesity Alters Placental and Umbilical Cord Plasma Oxidative Stress, a Cross-Sectional Study. Int. J. Mol. Sci..

[39] Clifton V.L. (2010). Sexual Dimorphism in the Human Placenta: Mediating Fetal Growth. Clin. Sci..

[40] Costa L.G., Vitalone A., Cole T.B., Furlong C.E. (2005). Paraoxonase (PON1): From Toxicology to Cardiovascular Medicine. Toxicol. Sci..

[41] Gabory A., Ferry L., Fajardy I., Jouneau L., Gothié J.D., Vige A., Jammes H. (2013). Maternal Diets and Stress Influence on Epigenetics and Sex-Specific Programming of Metabolism in the Offspring. Biochimie.

[42] Eriksson J.G., Kajantie E., Osmond C., Thornburg K., Barker D.J. (2010). Boys Live Dangerously in the Womb. Am. J. Hum. Biol..

[43] Shankar K., Harrell A., Kang P., Zheng Y., Zhao J., Ronis M.J.J. (2017). Maternal Obesity Promotes a Sex-Dependent Susceptibility to Metabolic Dysfunction in Adult Offspring. Clin. Sci..

[44] Dilek E., Kadıoğlu B.G., Nalçakan A. (2022). The paraoxonase 1 activity and lipid levels in umbilical cord blood and maternal venous blood, and their relations according to birth weight. Eur. Res. J..

[45] Stadler J.T., van Poppel M.N.M., Christoffersen C., Hill D., Wadsack C., Simmons D., Desoye G., Marsche G., Dali Core Investigator G. (2022). Gestational Hypertension and High-Density Lipoprotein Function: An Explorative Study in Overweight/Obese Women of the DALI Cohort. Antioxidants.

[46] Wang B., Jiang T., Qi Y., Luo S., Xia Y., Lang B., Zhang B., Zheng S. (2024). AGE-RAGE Axis and Cardiovascular Diseases: Pathophysiologic Mechanisms and Prospects for Clinical Applications. Cardiovasc. Drugs Ther..

[47] Nelson S.M., Matthews P., Poston L. (2009). Maternal metabolism and obesity: Modifiable determinants of pregnancy outcome. Hum. Reprod. Update.

[48] Kankowski L., Ardissino M., McCracken C., Lewandowski A.J., Leeson P., Neubauer S., Harvey N.C., Petersen S.E., Raisi-Estabragh Z. (2022). The Impact of Maternal Obesity on Offspring Cardiovascular Health: A Systematic Literature Review. Front. Endocrinol..

[49] Furigo I.C., Dearden L. (2022). Mechanisms mediating the impact of maternal obesity on offspring hypothalamic development and later function. Front. Endocrinol..

[50] Saben J., Lindsey F., Zhong Y., Thakali K., Badger T.M., Andres A., Gomez-Acevedo H., Shankar K. (2014). Maternal obesity is associated with a lipotoxic placental environment. Placenta.

[51] Al-Shajrawi O.M., Alwardat S., Alwardat N., Tengku Din T.A.D.A.A., Hussain Abdulrazak M., Musa I., Mussa A. (2024). Exploring the complex relationship between HIF-1 (rs11549465) and NFκB1 (rs28362491) variations and obesity (Review). World Acad. Sci. J..

